# An Electrophysiological Dissociation of Encoding vs. Maintenance Failures in Visual-Spatial Working Memory

**DOI:** 10.3389/fpsyg.2020.00522

**Published:** 2020-03-24

**Authors:** Jutta S. Mayer, Sebastian Korinth, Benjamin Peters, Christian J. Fiebach

**Affiliations:** ^1^Department of Psychology, Goethe University, Frankfurt, Germany; ^2^Department of Child and Adolescent Psychiatry, Psychosomatics and Psychotherapy, University Hospital Frankfurt, Goethe University, Frankfurt, Germany; ^3^Zuckerman Mind Brain Behavior Institute, Columbia University, New York, NY, United States; ^4^Institute of Medical Psychology, Goethe University, Frankfurt, Germany; ^5^Brain Imaging Center, Goethe University, Frankfurt, Germany

**Keywords:** working memory, encoding, maintenance, orientation, contralateral delay activity, N2

## Abstract

Working memory (WM) performance varies substantially among individuals but the precise contribution of different WM component processes to these functional limits remains unclear. By analyzing different types of responses in a spatial WM task, we recently demonstrated a functional dissociation between confident and not-confident errors reflecting failures of WM encoding and maintenance, respectively. Here, we use event-related brain potentials to further explore this dissociation. Healthy participants performed a delayed orientation-discrimination task and rated their response confidence for each trial. The encoding-related N2pc component was significantly reduced for confident errors compared to confident correct responses, which is indicative of an encoding failure. In contrast, the maintenance-related contra-lateral delay activity was similar for these response types indicating that in confident error trials, WM representations – potentially the wrong ones – were maintained accurately and with stability throughout the delay interval. However, contra-lateral delay activity measured during the early part of the delay period was decreased for not-confident errors, potentially reflecting compromised maintenance processes. These electrophysiological findings contribute to a refined understanding of the encoding and maintenance processes that contribute to limitations in WM performance and capacity.

## Introduction

Working memory (WM) allows us to actively hold and manipulate information in mind, thus making it available for a wide range of higher-order cognitive processes (Baddeley, 1986). A key characteristic of WM is its limited capacity (Luck and Vogel, 1997; Cowan, 2001) which varies among healthy young and older individuals (Todd and Marois, 2005; Cashdollar et al., 2013) and is substantially reduced in psychiatric disorders such as schizophrenia (Gold et al., 2010; Mayer et al., 2012). WM is viewed as emerging from the interplay between various component processes including encoding and maintaining information in WM (Bledowski et al., 2010; Eriksson et al., 2015), and therefore WM performance failures can occur due to different reasons. To understand the functional limits to WM performance and capacity, it is thus crucial to disentangle the component processes that interact during different stages of a WM task (Jonides et al., 2008).

The encoding of information into WM has received comparably less attention than processes of WM maintenance. However, increasing evidence from behavioral (Sperling, 1960; Schmidt et al., 2002; Fine and Minnery, 2009; Mayer et al., 2011; Robison et al., 2018), electrophysiological (Zanto and Gazzaley, 2009; Rutman et al., 2010; Gazzaley, 2011; Murray et al., 2011; Adam et al., 2015), and functional neuroimaging (Mayer et al., 2007; Fusser et al., 2011) studies suggests that attentional mechanisms facilitate WM encoding (Oberauer, 2019) – which might in turn prevent overloading of the capacity-limited WM system (Vogel et al., 2005; Fukuda and Vogel, 2009, 2011; Gaspar et al., 2016; Feldmann-Wüstefeld and Vogel, 2019). In addition, attentional mechanisms determine the precision with which a memory representation is formed (Bays and Husain, 2008; Huang and Sekuler, 2010; Bays et al., 2011). Furthermore, impaired early stage perceptual and attentional mechanisms contribute to reduced WM encoding and lower WM capacity for example in older persons (Gazzaley et al., 2005, 2008; Störmer et al., 2013) and in individuals with schizophrenia (Haenschel and Linden, 2011; Mayer et al., 2012).

One issue that complicates the investigation of failures in processes related specifically to WM encoding is the difficulty of isolating the encoding process in behavioral paradigms because performance measures (i.e., accuracy and reaction time) are compound measurements that potentially reflect processes associated with all different task phases (i.e., encoding, maintenance, and retrieval). In order to isolate WM encoding processes, we previously introduced a novel behavioral approach based on the analysis of different types of correct and erroneous responses depending on the trial-to-trial level of self-reported subjective response confidence (Lee et al., 2008; Mayer et al., 2011, 2014, 2018; Mayer and Park, 2012; Rademaker et al., 2012; Peters et al., 2019). Specifically, we reasoned that incorrect responses that were, however, given with confidence most likely reflect a problem at the encoding stage. Such “false memories,” according to this line of reasoning, arise as a result of erroneous encoding, which is, however, coupled with successful maintenance that nevertheless leads to a high confidence rating. In contrast, incorrect/not-confident (IN) responses are more likely caused by the degradation of representations during the active maintenance of WM contents, resulting in judgments of low confidence. Consistent with these assumptions, we have demonstrated a functional dissociation between confident and not-confident errors in the spatial delayed response task with different delay lengths (Mayer et al., 2018). In line with the encoding hypothesis, we have also shown that the percentage of incorrect/confident (IC) responses in a visuo-spatial delayed response task decreased when the processes that support WM encoding were facilitated (Mayer et al., 2011). Furthermore, this behavioral approach has been useful to dissociate encoding and maintenance deficits in patients with schizophrenia (Mayer and Park, 2012; Mayer et al., 2014, 2018).

So far, the neural mechanisms underlying confident and not-confident correct and incorrect responses in the spatial delayed response task are largely unknown. Using functional magnetic resonance imaging and near-infrared spectroscopy, one study reported similar delay-related activity in the prefrontal cortex for correct and IC responses in patients with schizophrenia (Lee et al., 2008). These neuroimaging findings support the assumption that confident errors do not reflect a failure of the active maintenance of WM contents. However, this study did not explicitly dissociate between neural activation related to WM encoding and WM maintenance.

**FIGURE 1:**
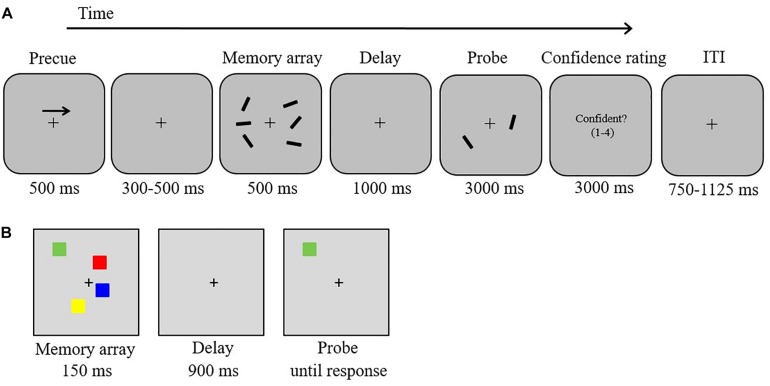
Stimuli and experimental procedure. **(A)** Delayed orientation-discrimination task used in the EEG experiment, **(B)** color change detection task.

In the present study, we took advantage of the high temporal resolution of electroencephalography (EEG) to explicitly track neural activity during WM encoding and maintenance in response to different response types. By using this approach, we sought to provide electrophysiological evidence for a functional dissociation of confident and not-confident errors in a spatial WM task and to elucidate the neural mechanisms that lead to functionally distinguishable failures of WM encoding and maintenance in healthy participants. EEG was recorded while participants performed a delayed orientation-discrimination task (Machizawa et al., 2012) that was followed by a rating of response confidence at the end of each trial (see Figure 1A). Combining these two responses resulted in four different types of trials, i.e., correct/confident (CC), CN, IC, and IN.

In EEG research, WM processes have often been studied using a change detection paradigm with lateralized presentation of the to-be-encoded stimuli. Participants are presented bilaterally with visual stimuli and are cued to remember the stimuli or stimuli features (e.g., color or orientation) on only one side of the display (Vogel and Machizawa, 2004; McCollough et al., 2007; Luria et al., 2016). The difference potential between electrodes contralateral and ipsilateral to the to-be-remembered information corrects for ERP effects reflecting low-level visual processes and local noise (which are bilateral because of the bilateral stimulus presentation) and isolates WM-specific ERP components (Luria et al., 2016). Using this approach, two lateralized components – the N2pc and the contralateral delay activity (CDA) – have been identified as indices of WM encoding and maintenance, respectively (Vogel and Machizawa, 2004; McCollough et al., 2007; Ikkai et al., 2010; Luria et al., 2016).

Specifically, the CDA is a slow negative voltage that emerges around 300 ms post onset of the to-be-encoded stimulus at posterior electrodes contralateral to the hemifield in which memory items were presented, and that persists throughout the delay interval. Its amplitude is sensitive to the number of items maintained in WM and correlates with the individual capacity limitation of WM (Vogel and Machizawa, 2004; Luria et al., 2016). Importantly, because the CDA amplitude is significantly reduced for incorrect trials relative to correct trials, it has been argued that such errors occur due to a failure of the active maintenance of WM contents (McCollough et al., 2007).

The N2pc component is an enhanced negativity observed contralateral to an attended stimulus over the posterior scalp about 200–300 ms after stimulus onset (Luck and Hillyard, 1994a, b). Numerous studies suggest that the N2pc component not only reflects the orienting of visual attention to select information for perceptual processing (Luck and Hillyard, 1994a, b; Eimer, 1996; Drew and Vogel, 2008; Hickey et al., 2009; Mazza et al., 2009) but also for WM encoding (McCollough et al., 2007; Ikkai et al., 2010; Störmer et al., 2013; Qi et al., 2014; Gaspar et al., 2016; Adam et al., 2018; Feldmann-Wüstefeld and Vogel, 2019). For example, it has been demonstrated that increasing the demands on target selection during the encoding period by lowering the stimulus contrast leads to a decrease in WM performance that is accompanied by a reduction in the N2pc amplitude (Ikkai et al., 2010). Some evidence suggests that the amplitude of the N2pc predicts WM capacity in young adults (Störmer et al., 2013; but see also Gaspar et al., 2016; Feldmann-Wüstefeld and Vogel, 2019) and is reduced during WM encoding in older adults (Störmer et al., 2013). The N2pc component, thus, seems to be a valuable tool for studying the role of attentional processes during WM encoding.

Our hypotheses focused on the N2pc and CDA components as well-established ERP indicators of WM encoding and maintenance. Given the association between N2pc and attentional processes during WM encoding (Ikkai et al., 2010), we expected a reduction of N2pc amplitude specifically in IC trials relative to CC trials. This would support the hypothesis that IC responses reflect inefficient WM encoding due to reduced attentional selection (Hypothesis 1). Also, if IC responses reflected a problem at the encoding stage rather than difficulties in maintaining a stable (albeit wrong) WM representation, CDA amplitudes should not differ between CC and IC responses (Lee et al., 2008). In contrast, the CDA amplitude should decrease in IN trials, reflecting a degradation of representations during the active maintenance of WM contents (McCollough et al., 2007) (Hypothesis 2).

Following previous research on WM capacity constraints (Vogel and Machizawa, 2004; Vogel et al., 2005; Fukuda et al., 2015; Gaspar et al., 2016; Feldmann-Wüstefeld and Vogel, 2019), we also asked whether reduced attentional selection during WM encoding would predict individual differences in WM capacity (as opposed to performance in the experimental WM task; Exploratory Research Question). To investigate this, we correlated WM capacity estimates derived from an independent visual change detection task (Figure 1B) with behavioral indices and ERP components from the delayed orientation-discrimination task.

Lastly, we also performed two behavioral control experiments in order to exclude that performance in the delayed orientation-discrimination task could be attributed either to deficits in perceptual processing (i.e., the sensory control task) or encoding speed (i.e., the encoding control task).

## Materials and Methods

### Participants

Forty-seven students from Goethe University, Frankfurt participated for course credit or monetary reimbursement. Participants reported normal or corrected-to-normal visual acuity and no history of neurological or psychiatric illness. All participants gave written informed consent. The study protocol was approved by the Ethical Board of the Medical Faculty, Goethe University, Frankfurt. Three participants were excluded from the study due to low behavioral performance (mean response accuracy >2 SD below the group mean) in either the sensory control task (two participants) or the encoding control task (one participant) (see section “Experimental Tasks and Procedure”). Therefore, the final sample of the study consisted of 44 participants (24 males; mean age = 23.07, SD = 4.80; mean IQ^1^ = 104.79, SD = 14.51; all right-handed). Sample sizes for the analyses of behavioral data and ERPs can nevertheless vary further due to different reasons (see the descriptions of the specific experimental tasks for further details). Most importantly, for our main hypothesis test, data of three participants were not entered into the ERP analyses because of a low number of IC responses (range: 0–9 trials), which is the critical experimental condition for evaluating our hypotheses. After artifact rejection, data of additional five participants were excluded because of a low number of trials for either of the response types (range: 3–16 trials, see section “Electrophysiological Recordings and Analysis”). Therefore, the final ERP analyses were based on data of 36 participants (males: 17; mean age = 23.22, SD = 5.04; mean IQ = 106.66, SD = 15.21; all right-handed).

### Experimental Tasks and Procedure

The study consisted of three consecutive sessions on separate days. The delayed orientation-discrimination task was the main task that was used in the EEG experiment, conducted during sessions 2 and 3. During the first session, participants (a) performed two behavioral control experiments that also served to familiarize them with the stimuli and task used in the EEG experiment, (b) conducted a color change detection task to behaviorally assess WM capacity (Mayer et al., 2012), and (c) completed a brief IQ test (Lehrl, 1999). Total duration of the first session was 60 min.

#### Delayed Orientation-Discrimination Task

In an electromagnetically shielded room, participants were positioned on a head-and-chin rest at a viewing distance of 120 cm from a 19-inch monitor (60 Hz; 1,920 × 1,080 screen resolution). The software Presentation (Neurobehavioral Systems) was used to create and execute the experiment, and manual responses were registered via keyboard button presses. Trial displays were presented on a gray background (RGB values: 150, 150, and 150), with a centrally presented black fixation cross (0.19° width) appearing constantly throughout each block. In each trial (Figure 1A), participants were presented with a brief bilateral array of black bars (length: 1.15°; three bars per hemifield) of varying orientations. Each bar was randomly (without replacement) assigned one of 12 orientations (ranging from 5° to 170° in intervals of 15°). The two stimulus arrays were presented within 7.0° × 7.3° rectangular regions that were presented 3.0° to the left and right of the central fixation cross. The position of the bars inside the bilateral regions was randomized, with the constraint that the distance between bars within a hemifield was at least 2.2° (center-to-center). The task was to remember the orientations of the three bars in either the left or the right hemifield.

Each trial started with a 500-ms central arrow that appeared 2° above the fixation cross (Figure 1A). The arrow cued participants to remember the orientations of the three bars in either the left or the right hemifield of the memory array (50% left). Following a variable interval of 300–500 ms, a memory array was presented for 500 ms. The memory array was removed from the display for 1,000 ms (retention period). A test array was then displayed for 3,000 ms, containing in each hemifield one of the three bars presented in the memory array. In each hemifield, the location of the test bar was randomly chosen from the three locations of the memory set. The bar in the cued hemifield was rotated by 45° clockwise or counterclockwise (equiprobable across trials) relative to the corresponding bar in the memory array. Participants indicated by a button press whether the bar was rotated clockwise (right button, “L” on the keyboard) or counterclockwise (left button, “K” on the keyboard)^2^. Participants were instructed to use the right index finger to press the left button (labeled with an arrow rotating counterclockwise) and the right middle finger to press the right button (labeled with an arrow rotating clockwise). Immediately after the decision, participants rated the confidence level for their response on a scale from 1 (not confident at all) to 4 (very confident) by pressing the buttons “A” (labeled “1”), “S” (labeled “2”), “D” (labeled “3”), or “F” (labeled “4”) with the left index finger. An inter-trial interval (750–1,125 ms) followed. The instructions emphasized accuracy rather than speed. Moreover, participants were also instructed to keep their eyes fixated throughout the task. Participants participated in two EEG sessions (total duration including breaks: 60 min per session) on two consecutive days. Each session consisted of one practice block (10 trials) followed by six experimental blocks of 52 trials each, yielding a total of 624 trials (312 clockwise rotation, 312 counterclockwise rotation). The experimental factors rotation direction (clockwise vs. counterclockwise) and cued side (left vs. right) were pseudo-randomly intermixed within each block with the constraint that each block contained the same number of trials for each of the four possible combinations (13 trials per combination). We quantified the percentage of type of responses depending on the trial-to-trial level of self-reported subjective response confidence (CC, CN, IC, and IN). To obtain a sufficient number of confident and not-confident error trials for ERP analyses, confident and not-confident responses were defined as responses that were given with confidence ratings of 3 or 4 vs. 1 or 2, respectively. To assess whether potential performance differences occurred due to clockwise vs. counterclockwise rotation changes, we also analyzed mean response accuracy as a function of direction of rotation change using *t*-statistics (two-tailed). For all analyses we report exact *p*-values and Bonferroni corrected thresholds if appropriate.

#### Sensory Control Task: Orientation-Discrimination Task Without Delay

The sensory control task was implemented in order to assess deficits in perceptual processing independent of WM demands. To this end, the same task, stimuli, and procedure were used as in the delayed orientation-discrimination task but memory requirements were minimized. We reasoned that if deficits in the perceptual processing and discrimination of the orientation stimuli occurred, such deficits would also contribute to reduced performance in the delayed orientation-discrimination task. In this case, the different types of errors would not solely reflect failures of WM encoding or maintenance. To minimize the contribution of perceptual processing deficits to reduced performance in the delayed orientation-discrimination task, participants with low performance in the sensory control task were excluded from the entire study.

The test array appeared immediately after the memory array without a retention interval, and participants indicated by a button press whether the bar presented in the test array was rotated clockwise or counterclockwise relative to the corresponding bar in the preceding array. Note that the presentation of the test immediately after the memory array might have induced an impression of apparent motion that is highly unlikely to occur in the main experiment, i.e., in the delayed orientation-discrimination task. This potential confound needs to be taken into account when comparing behavioral performance across tasks. However, the sensory control task predominantly served to exclude participants with very low visual discriminating abilities – a process that was required to correctly indicate the rotation direction. Response times were not limited. Participants performed one practice block (10 trials) followed by two blocks of 40 trials each. The experimental factors rotation direction (clockwise vs. counterclockwise) and cued side (left vs. right) were pseudo-randomly intermixed within each block with the constraint that each block contained the same number of trials for each of the four possible combinations (10 trials per combination).

Mean response accuracy was analyzed as a function of direction of rotation change (clockwise vs. counterclockwise) using *t*-statistics (two-tailed). Two participants with mean response accuracies >2 SD below the group mean (participant 1: 53.8% correct; participant 2: 57.5% correct) were excluded from the entire study (see section “Participants”).

#### Encoding Control Task: Delayed Orientation-Discrimination Task With Variable Encoding Lengths

Inter-individual differences in perceptual processing speed is another factor that might influence task performance and ERP amplitudes in the delayed orientation-discrimination task (Wiegand et al., 2014), thus making it difficult to assess behavioral and ERP indices reflecting failures of WM encoding and maintenance independent from limitations in perceptual processing. This is specifically problematic when implementing a single, constant presentation time of the to-be-encoded stimulus array. In the EEG task, the encoding period was 500 ms. To ensure that failures of WM were not mainly due to insufficient encoding time, we ran a control experiment in which we additionally included also trials with an encoding period of 1,000 ms. In this control experiment, we tested the effect of encoding length on WM performance and on the distribution of response types. We reasoned that if task performance and the distribution of response types did not significantly differ as a function of encoding length, performance indices should largely reflect limitations in WM encoding and maintenance rather than limitations in perceptual performance (even at the relatively short encoding length of 500 ms).

The same task, stimuli, and procedure were used as in the main experiment (delayed orientation-discrimination task with memory demand), with two exceptions: In half of the trials, the exposure time of the memory array was extended to 1,000 ms. In addition, response times were not limited in this task. Participants performed one practice block (10 trials) followed by two blocks of 40 trials each (20 trials with 500 ms-encoding length and 20 trials with 1,000 ms-encoding length, randomly intermixed), yielding a total of 80 trials. Rotation direction (clockwise vs. counterclockwise) and cued side (left vs. right) were pseudo-randomly intermixed within each block with the constraint that each block contained the same number of trials for each of the four possible combinations (10 trials per combination).

Mean response accuracy was analyzed as a function of direction of rotation change (clockwise vs. counterclockwise) and encoding length (500 ms vs. 1,000 ms) using a repeated-measures analysis of variance (ANOVA). One participant with mean response accuracy >2 SD below the group mean (i.e., 52.5% correct) was excluded from the entire study (see section “Participants”). Due to technical problems data from one further participant was not correctly recorded and thus excluded from the analysis of the encoding control task (*N*_enc_control_ = 43). Because valid data for the other tasks (EEG task and sensory control task) was available, this participant was included in all other analyses.

#### Color Change Detection Task

A standard color change detection task (Vogel and Machizawa, 2004; Mayer et al., 2012; Figure 1B) was used in order to estimate individuals’ WM capacity and to correlate these estimates with behavioral and ERP indices derived from the delayed orientation-discrimination task.

Stimuli in the change detection task were colored (red, green, blue, yellow, purple, black, and white) squares (1.2° × 1.2°). The squares were presented in randomly selected positions within a centered region (11.4° × 11.4°) on a gray background (RGB values: 125, 125, and 125).

In each trial, participants were presented with arrays of two, four, six, or eight colored squares for 150 ms (memory array). After a retention interval of 900 ms, one colored square (test probe) was presented at the location of one of the items from the memory array. Participants made an unspeeded button press to indicate whether the color of the test probe matched or did not match the color of the original memory item in that location. Half of the trials were matches. An inter-trial interval of 1 s followed. Each of the four load conditions was presented equally often (40 trials per condition). Participants performed 10 practice trials, followed by an experimental block of 160 trials (which were presented in a randomized order).

To quantify the individual WM capacity we used an equation developed by Pashler (1988) and modified by Cowan (2001): *K* = (hit rate + correct rejection rate−1) × *N*. This approach allows us to estimate the number of items held in memory, *K*, from an array size of *N* items, taking guessing into account^3^. The *K* estimate is conceptualized as a limit in the number of discrete slots that holds a single item, which is appropriate for the change detection tasks with highly distinguishable stimuli, such as categorically different colors (Rouder et al., 2011). The *K* estimate has become a standard measure of change detection performance because it corrects for response bias and allows comparisons across different array sizes and conditions (Luck and Vogel, 2013). We first transformed each participant’s accuracy for each array size (2, 4, 6, and 8) into an estimate of *K* as index of individual WM capacity. For each participant, we then calculated the mean *K* value across the four array sizes. Data from one participant of the final EEG sample of 44 participants was missing (*N*_capacity_task_ = 43).

### Electrophysiological Recordings and Analysis

**FIGURE 2:**
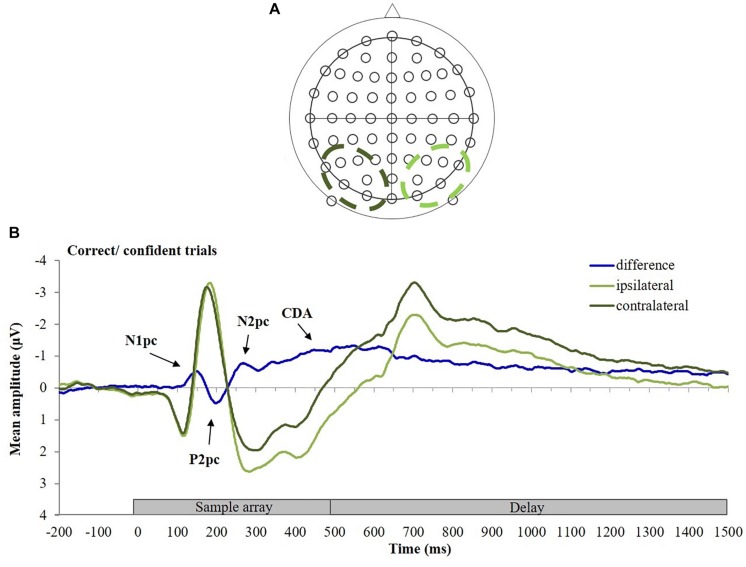
**(A)** Arrangement of electrodes, **(B)** calculation of the contralateral difference wave. ERPs elicited at posterior electrodes contralateral (dark green) and ipsilateral (light green) to the cued stimuli (in this example the participant was cued to the stimuli in the right hemifield of the memory array), and the resulting difference wave (blue) for correct/confident trials are shown.

EEG was recorded continuously from 64 active Ag/AgCl scalp electrodes mounted on an elastic cap and amplified by a BrainAmp amplifier (Brain Products, München, Germany). Electrodes were arranged according to the extended 10–10 system (Figure 2A). The horizontal electrooculogram was recorded from a pair of electrodes placed lateral to the external canthi, and the vertical electrooculogram was recorded from an electrode placed below the left eye and referenced to the Fp1 electrode. All signals were recorded with a bandpass of 0.1–200 Hz (without a notch filter) and electrodes were digitized at a sampling rate of 1,000 Hz, referenced online to the FCz electrode and grounded to AFz. The BrainVision Analyzer 2.0 software (Brain Products, München, Germany) was used for offline data analyses.

As already described above, data from three participants were excluded from the EEG analysis due to a low number of IC trials (<10 trials, see section “Participants”), leaving 41 participants for EEG analysis. Continuous EEG data were filtered offline with a 0.1 Hz (12 dB/oct) high-pass filter, a 30 Hz (12 dB/oct) low-pass filter, and a 50 Hz notch filter. Blinks and eye-movements were corrected using the method of Gratton et al. (1983). Preprocessed data were re-referenced to the average of all scalp electrodes. (Note that while many CDA studies use linked mastoids as reference, we here preferred an average reference as the mastoid electrodes are physically very close to the occipital electrodes which are relevant for our study due to our focus on encoding-related WM processes). The two data sets for each participant were combined and epochs time-locked to the onset of the memory array were extracted (epoch length: −200 to 1,500 ms relative to the onset of the memory array). Epochs containing artifacts were rejected; this included channel blockings (lowest allowed activity in intervals of 100 ms: 0.5 μV, 200 ms before and after an event) and bad gradients (maximally allowed voltage step: 50 μV/ms, 200 ms before and after an event). Participants with trial rejection rates of 45% and above due to eye movements and muscle artifacts across all channels were excluded from further data analyses. This resulted in exclusion of five participants – each of them having a low number of trials (range: 3–16 trials) for at least one of the response types. For the remaining 36 participants, artifact rejection was done individually at each electrode. For theses participants an average of 98% (SD = 3.77%, range 88–100%) of all measured trials were included in the data analyses, leading to at least 26 trials for each response type per subject [mean number of trials averaged across the 12 posterior electrodes used for analysis (see next paragraph): CC = 353 (SD = 75.03, range = 193–516), CN = 98 (SD = 52.91, range = 26–239), IC = 87 (SD = 41.30, range = 26–169), and IN = 72 (SD = 30.84, range = 30–145)].

ERPs were averaged, separately for each participant and condition, and normalized relative to the 200 ms baseline time window. ERPs were then collapsed across homologous lateral positions of the electrodes (left vs. right) and across the to-be-remembered hemifields (left vs. right) to obtain waveforms from electrodes located contralateral vs. ipsilateral to the to-be-remembered stimuli. Mean amplitude differences were computed by subtracting ipsilateral from contralateral ERPs averaged from six pairs of posterior electrode sites (P3/P4, P5/P6, P7/P8, PO3/PO4, PO7/PO8, and O1/O2; see Figure 2A), following previously published procedures (Vogel et al., 2005; Machizawa et al., 2012; Störmer et al., 2013; Qi et al., 2014; Xie and Zhang, 2018). To demonstrate the calculation of the contralateral difference ERP, Figure 2B displays the ERPs elicited at posterior electrodes contralateral (dark green) and ipsilateral (light green) to the stimulus, and the resulting difference wave (blue) for the CC trials. To ensure that contralateral effects occurred at all electrode pairs, a prerequisite for averaging across electrode pairs, we also computed lateralized ERPs at individual electrodes pairs (see Supplementary Material 1 and Supplementary Figure 1).

To assess encoding-related activity, we calculated the N2pc component during a measurement window of 230–290 ms relative to the onset of the memory array. This narrow time window was chosen following a previous study that measured mean amplitude (Gaspar et al., 2016), with the aim of dissociating the N2pc component from earlier sensory components (see section “Early Visual Components”). The CDA was calculated during two time-windows, i.e., 400–1,000 ms (early CDA component), and 1,000–1,500 ms (late CDA component) relative to stimulus onset. Because the CDA is known to decrease toward the end of the delay period due to processes related to the anticipation of the test array (McCollough et al., 2007), we focused on the early CDA component.

Hypothesis 1 explicitly referred to the N2pc amplitude difference between CC and IC responses, which was tested using a planned paired *t*-test (one-tailed) due to the directional nature of the hypothesis. Hypothesis 2 referred to CDA amplitude differences and consisted of two parts. First, CDA amplitude differences between CC and IC responses were tested with a two-tailed *t*-test due to the non-directional nature of the hypothesis. Null-results with regard to these planned *t*-tests were additionally statistically evaluated by conducting Bayesian undirected one sample *t*-tests (Rouder et al., 2009, 2012) using the JASP statistical software (JASP Team, 2019). Bayes factors (BF_01_) derived from these analyses were reported as the natural logarithm of the odds of the null hypothesis (H_0_) over the alternative hypothesis (H_1_). For Bayesian *t*-tests, we used the default prior on effect size (Cauchy distribution, centered on zero, with rate *r* = 0.707). Second, CDA amplitude differences between IC and IN responses were tested with a one-tailed *t*-test due to the directional nature of the hypothesis.

For all components (N2pc, early CDA, and late CDA), normal distribution of the difference values (CC vs. IC, IC vs. IN) was confirmed (Kolmogorov–Smirnov test, all *p*-values >0.08). No extreme outliers were observed. Cohen’s *d* was used to indicate effect sizes.

Inherent to our paradigm, participant-specific variations in the amount of correct and incorrect responses and confidence ratings generated an unbalanced distribution of available data points (i.e., trials) across participants in each of the four response conditions. To address this problem, N2pc and CDA components were also analyzed using linear mixed-effect models (LMM; Kliegl et al., 2010) that explicitly model individual differences as random effects. Results of these analyses are reported as Supplementary Material (see Supplementary Material 5 and Supplementary Table 3).

#### Early Visual Components

During WM encoding, the N2pc was preceded by lateralized early stage sensory components (N1pc, P2pc; see Figure 2B) which are known to index early perceptual processing (Hillyard et al., 1998) and attentional evaluation of the significance of visual stimuli (Straube and Fahle, 2010) – processes that might also be relevant for efficient and/or precise WM encoding (Schmidt et al., 2002; Fine and Minnery, 2009; Gazzaley, 2011). Because these components have been studied only rarely in the context of the lateralized visual change detection task (Störmer et al., 2013), we formulated no specific hypotheses but analyzed N1pc (130–170 ms) and P2pc (180–220 ms) components in response to the four different trials types in an explorative way. We used separate within-subject two-way ANOVAs including the factors response correctness (correct vs. incorrect) and response confidence (confident vs. not confident). Normal distribution of difference values was confirmed for all contrasts (Kolmogorov–Smirnov test, all *p*-values >0.067) except for the N1pc contrast of CN vs. IN (*p* = 0.035).

### Correlations With WM Capacity Estimates

To better understand the functional limits to WM capacity, we also performed correlational analyses. Specifically, we asked whether failures of WM encoding and/or WM maintenance would predict individual differences in WM capacity. To this end we correlated individual mean *K* values (see section “Color Change Detection Task”) with the subject-specific amount of IC responses (as an indicator of reduced attentional selection during WM encoding) as well as IN responses (as an indicator of impaired WM maintenance) using Spearman’s correlation coefficient (Kolmogorov–Smirnov test, *p* = 0.04 for mean *K* values, *N* = 43). With regard to ERPs, we calculated for each participant the mean N2pc amplitude difference between CC and IC trials as index of reduced attentional selection during incorrect WM encoding and correlated this subject-specific difference value with the individual mean *K* values using Pearson’s correlation coefficient. Furthermore, we calculated for each participant the mean early CDA amplitude difference between IC and IN responses as an index of aberrant WM maintenance and correlated this subject-specific difference value with the individual mean *K* values using Pearson’s correlation coefficient. Normal distribution was given for mean *K* values (Kolmogorov–Smirnov test, *p* = 0.09, *N* = 36).

## Results

### Behavioral Results

#### EEG Orientation Change Detection Task

**FIGURE 3:**
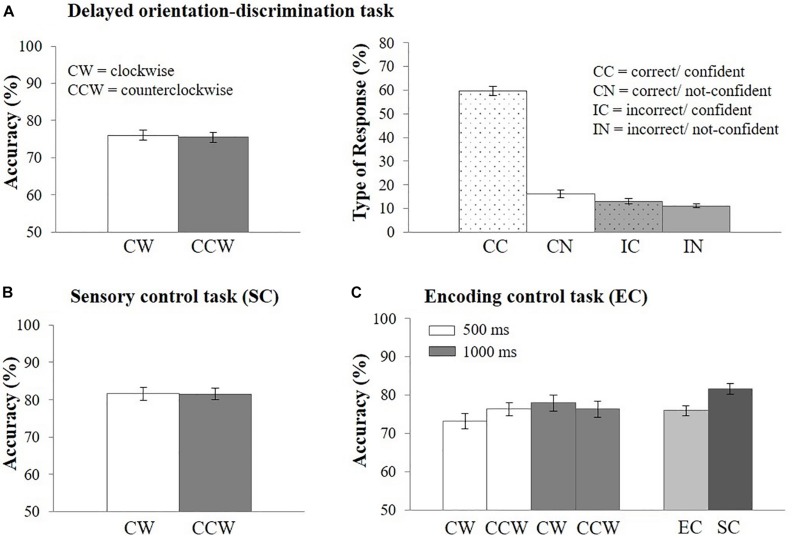
Behavioral results. **(A)** Response accuracy (left side) and percentage of type of response (right side) in the EEG delayed orientation-discrimination task, **(B)** response accuracy in the sensory control task (SC), **(C)** response accuracy in the encoding control task (EC) and in comparison with the sensory control task (SC). Error bars represent the standard error of the mean. CW, clockwise; CCW, counterclockwise; CC, correct/confident; CN, correct/not-confident; IC, incorrect/confident; IN, incorrect/not-confident.

Mean response accuracy was *M* = 75.76% (*N* = 44, SD = 8.22, range = 62.66–96.79%). Response accuracy did not vary as a function of the direction of orientation change on the retrieval probe [i.e., clockwise vs. counterclockwise; *t*(43) = 0.46, *p* = 0.65; Cohen’s *d* = 0.07, see Figure 3A]. Overall, CC responses occurred most often (*M* = 59.55% of all trials, SD = 13.38, range: 23.06–85.36%). 16.26% (SD = 10.49, range: 4.6–58.73%) of trials were CN responses, 13.08% (SD = 7.31, range: 0–27.41%) were IC responses, and 11.11% (SD = 4.99, range: 3.2–23.43%) were IN responses (Figure 3A). Response type distributions were also analyzed for high-performing (*N* = 22, *M* = 82.39, SD = 5.61) and low-performing (*N* = 22, *M* = 69.13, SD = 3.83) participants using a median split based on the mean accuracy in the delayed orientation-discrimination task (see Supplementary Material 2 and Supplementary Table 2).

#### Behavioral Control Tasks

Mean response accuracy was highest in the sensory control task (*M* = 81.56%, SD = 9.23, range = 61.3–98.8%; Figure 3B) and decreased when memory requirements were added [encoding control task: *M* = 75.95%, SD = 8.87, range = 59.5–97.5%; *t*(42) = 3.21, *p* < 0.01, Cohen’s *d* = 0.49, Figure 3C]. Importantly, response accuracy did not vary as a function of direction of orientation change (clockwise vs. counterclockwise), neither in the sensory control task, [*t*(43) = 0.03, *p* = 0.97, Figure 3B], nor in the encoding control task [Figure 3C, main effect of direction of orientation: *F*(1,42) = 0.11, *p* = 0.74]. Thus, the demands on perceptual discrimination were comparable for clockwise and counterclockwise orientation changes. In addition, response accuracy differed only descriptively, but not statistically, between encoding lengths of 1,000 ms (*M* = 77.14%, SD = 10.67) vs. 500 ms [(*M* = 74.77%, SD = 8.97), *F*(1,42) = 3.29, *p* = 0.08, Figure 3C]. The interaction between response orientation and encoding length was also not significant [*F*(1,42) = 2.08, *p* = 0.16]. We also analyzed the percentage of type of responses and found similar distributions across encoding lengths (Supplementary Material 3) and tasks (Supplementary Material 4). Because task performance and the distribution of responses types were comparable across encoding lengths we reasoned that the encoding period of 500 ms implemented in the EEG task was sufficient to perceive the stimuli.

#### Color Change Detection Task

Mean WM capacity averaged across the four array sizes was *M* = 2.65 (SD = 0.57, range: 1.78–4.13). This is comparable to findings of previous experiments using this paradigm (Vogel et al., 2005; Fukuda and Vogel, 2011). To test whether individual differences in WM capacity can be directly related to failures of WM encoding and/or maintenance at the behavioral level, we correlated individual mean *K* values with the subject-specific amount of IC responses (as an indicator of reduced attentional selection during WM encoding) as well as IN responses (as an indicator of impaired WM maintenance). WM capacity estimates correlated negatively with the percentage of IC responses (*r* = −0.34; *p* = 0.024) but not the percentage of IN responses (*r* = −0.05, *p* = 0.76) derived from the main experiment (orientation change detection task conducted in the EEG; Bonferroni corrected statistical threshold for two tests: *p* = 0.025).

### Event-Related Brain Potentials

**FIGURE 4:**
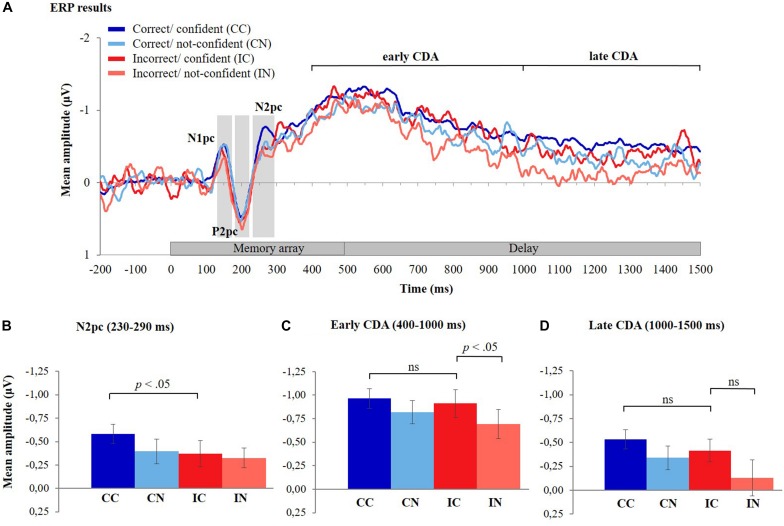
ERP results. **(A)** The grand-averaged waveforms for the four different response types, timelocked to memory array onset. The contralateral minus ipsilateral difference waves averaged across six posterior electrode pairs (P3/P4, P5/P6, P7/P8, PO3/PO4, PO7/PO8, and O1/O2) are shown during the presentation of the stimuli (0–500 ms) and the delay period (500–1,500 ms). The grand-averaged waveform for correct/confident responses was less noisy compared to the other three response types due to the higher number of trials for this response type (mean number of trials across participants: CC = 353, CN = 98, IC = 87, and IN = 72). **(B)** Mean amplitudes of the N2pc component (230–290 ms after memory array onset) for the different response types, **(C)** mean amplitudes of the early CDA component (400–1,000 ms after memory array onset), and **(D)** late CDA component (1,000–1,500 ms after memory array onset) for the different response types. Error bars represent standard errors of the means. CDA, contralateral delay activity.

Figure 4A depicts ERP difference waveforms for the four different response types (CC, CN, IC, and IN) averaged across six posterior electrode pairs (see section “Materials and Methods” and Figure 2A for details). (Note that due to the lower number of trials, the ERPs for CN, IC, and IN responses are less smooth than those for CC responses; compare also to Figure 2B). During the encoding phase (i.e., 0–500 ms after memory array onset), a negative deflection peaking at about 150 ms after onset of the memory array (N1pc) was followed by a positive deflection peaking at around 200 ms (P2pc) and another negative deflection peaking around 270 ms (N2pc). This pattern was observed for all response types. The CDA difference waveforms started to increase around 300 ms after memory array onset and were observed throughout the delay phase (500–1,500 ms after memory array onset). ERP amplitude differences between response types are described statistically in the following sections.

#### Hypothesis 1: Effect of Response Type on the N2pc

The mean amplitude of the N2pc component was largest for CC responses and considerably lower for all other response types (Figure 4B). Consistent with Hypothesis 1, the mean amplitude of the N2pc component was significantly lower for IC responses compared to CC responses [*t*(35) = −2.18, *p* = 0.018, one-tailed, Cohen’s *d* = 0.36] (see Supplementary Material 5 and Supplementary Table 3 for findings from LMMs).

#### Hypothesis 2: Effect of Response Type on the Early and Late CDA

Hypothesis 2 consists of two parts, i.e., (a) that CDA amplitudes do not differ between CC and IC responses (which was tested using a two-sided *t*-test) and (b) that CDA amplitudes decrease in IN relative to IC trials (examined using a one-sided *t*-test due to the directional nature of the hypothesis). Consistent with these hypotheses, we observed that mean amplitudes of the early CDA did not significantly differ between CC and IC responses [*t*(35) = −0.62, *p* = 0.539] (Figure 4C). Calculation of the Bayes factor (BF_01_) yielded 4.67 times stronger support for the null hypothesis of no difference between CDA amplitudes of CC and IC responses, over the alternative hypothesis (i.e., of the existence of a difference in CDA amplitude between CC and IC trials). In contrast, the early CDA was significantly lower for IN responses compared to IC responses [*t*(35) = −2.27, *p* = 0.015, one-tailed, Cohen’s *d* = 0.38, Bonferroni corrected threshold for two tests: *p* = 0.025] (also see Supplementary Material 5 and Supplementary Table 3 for findings from LMMs).

Similarly, the mean amplitude of the late CDA did not differ significantly between IC and CC responses [*t*(35) = −1.177, *p* = 0.247] and computation of the Bayes factor (BF_01_) showed 2.955 times stronger support for the null hypothesis of no difference between CC and IC responses over the alternative hypothesis. The mean amplitude of the late CDA was, however, not significantly lower for IN responses compared to IC responses [*t*(35) = −1.987, *p* = 0.0274, one-tailed, Cohen’s *d* = 0.33, Bonferroni corrected threshold for two tests: *p* = 0.025] (Figure 4D, also see Supplementary Material 5 and Supplementary Table 3 for findings from LMMs).

#### Exploratory Analysis: Effect of Response Type on Early Visual Components

ERP components indexing perceptual processes before the N2pc have only rarely been studied in the context of lateralized WM tasks, which is why we had not specified hypotheses *a priori*. We nevertheless examined possible modulations by trial type in an exploratory manner using within-subject two-way ANOVAs with the factors response correctness (correct vs. incorrect) and response confidence (confident vs. not confident), separately for the N1pc and P2pc components. However, we observed no significant main or interaction effects on the N1pc (all *p*-values ≥0.10) or on the P2pc (all *p*-values ≥0.22).

#### Exploratory Analysis: Correlation With WM Capacity Estimates

Individual WM capacity estimates neither correlated with the subject-specific mean reduction of the N2pc amplitude for IC vs. CC responses (*r* = 0.12, *p* = 0.50) nor with the subject-specific mean reduction of the early CDA amplitude for IN vs. IC responses (*r* = −0.13, *p* = 0.45).

## Discussion

The present study provides important insights into the role of encoding and maintenance processes for failures in WM performance, by analyzing electrophysiological correlates of different types of responses in the delayed orientation-discrimination task in healthy participants. Our key findings derived from *t*-statistics are (i) that during WM encoding the target-related N2pc component was significantly reduced for IC responses compared to CC responses, whereas, (ii) the maintenance-related CDA component was similar for correct and incorrect responses when given with high confidence. (iii) In contrast, the amplitude of the early CDA was significantly reduced for IN responses relative to IC responses. However, the amplitude of the late CDA was not significantly reduced for IN responses compared to IC responses. Together, these ERP findings suggest a functional dissociation between confident and not-confident errors in the delayed orientation-discrimination task, with IC responses reflecting inefficient WM encoding (supporting Hypothesis 1) and not-confident errors most likely reflecting inefficient WM maintenance (supporting Hypothesis 2). This is in line with the previous behavioral evidence derived from studies that manipulated either the delay length (Mayer et al., 2018) or encoding demands (Mayer et al., 2011) in the spatial delayed response task. In addition, these findings are consistent with previous neuroimaging results (Lee et al., 2008) indicating that confident errors are not due to failures during the active maintenance of WM contents. However, the previous study did not assess neural activity associated with WM encoding, because functional magnetic resonance imaging lacks the temporal resolution necessary to resolve between encoding and maintenance processes. By combining the analysis of different types of responses depending on the trial-to-trial level of self-reported subjective response confidence with electrophysiological measurements, we were in the present study able to disentangle the contribution of encoding and maintenance processes to the functional limits of WM performance.

### The Contribution of WM Encoding and Maintenance Failures to Limitations in WM Performance

#### What Are the Processes That Explain Failures of WM Maintenance?

The CDA, which persists throughout the delay interval, has been widely accepted as an electrophysiological marker of the active maintenance of visual WM contents (Vogel and Machizawa, 2004; Luria et al., 2016). In the present study the amplitude of the early CDA did not significantly differ between CC and IC responses but was reduced for IN responses. In the light of previous studies that have associated a reduction of the CDA amplitude for incorrect/poor performance trials relative to correct/good performance trials with a failure of WM maintenance (McCollough et al., 2007; Adam et al., 2018), the amplitude reduction of the early CDA associated with IN responses observed in our study may be best interpreted in terms of compromised maintenance processes that contribute to reduced WM performance. However, this CDA reduction was not consistently found in the present sample (i.e., the effect was found for the early but not the late CDA and it was not found when calculating LMMs), and therefore interpretations are only preliminary. Importantly, early CDA amplitudes were reduced only when incorrect responses were given with low confidence. As suggested by behavioral findings, response confidence predicts the precision of visual WM representations (Rademaker et al., 2012; Peters et al., 2019). In addition, it has been shown that the CDA amplitude reflects not only the number of stored items but also the precision with which items are maintained especially when the number of items is small (Machizawa et al., 2012). Therefore, not-confident errors associated with low early CDA amplitudes in our study might reflect reduced precision with which orientations were maintained, leading in turn to WM performance failures. Most importantly, we found no evidence for a reduction of early and late CDA amplitudes for trials with IC responses relative to those with CC responses. (Note, however, that analyses of Bayes factors for these null results did not provide strong evidence for the null hypothesis, so that these results should be replicated in a study with more statistical power). In contrast, during WM encoding, the N2pc amplitude was reduced for IC vs. CC responses, which indicates a failure of WM encoding. Together, these findings suggest that despite erroneous encoding, WM representations, potentially the wrong ones (i.e., “false memories”), were maintained accurately and with stability throughout the delay interval in IC trials. Interestingly, these findings are in line with predictions from a neurocomputational network model of spatial WM deficits in schizophrenia that has linked synaptic alterations in prefrontal circuits to specific types of errors that are conceptually very similar to the confident and not-confident errors as defined in our behavioral study (Cano-Colino and Compte, 2012). Specifically, in this model not-confident errors were characterized by a decay in the stimulus-specific network activity by the end of the delay period reflecting decreased stability of the WM representation during the delay phase. In contrast, confident errors were associated with spontaneous activity representing random spatial locations which emerged in the encoding phase of the task and remained high throughout the delay period. Thus, reduced WM performance cannot in every case be explained solely by the loss of information during the delay period. Rather, these findings suggest that cognitive and neural models of WM need to take into account that reduced WM performance can also result from inefficient processes during WM encoding.

#### What Are the Processes That Explain Inefficient WM Encoding?

The N2pc component is a well-established marker of the deployment of attention to relevant information in visual space (Luck and Hillyard, 1994a, b; Eimer, 1996). Recent evidence suggests that the N2pc reflects the selective enhancement of the cortical representation of attended items (Hickey et al., 2009; Mazza et al., 2009; Gaspar et al., 2016) rather than the suppression of unattended items (Luck and Hillyard, 1994b). In the context of WM encoding, the attentional prioritization of targets might increase the precision with which a memory representation is formed (Bays and Husain, 2008; Huang and Sekuler, 2010; Bays et al., 2011). In line with this assumption, it has been found that the target-related N2pc component was reduced when the contrast of the to-be-encoded stimuli was decreased, whereas the CDA was not affected (Ikkai et al., 2010). Moreover, we have previously shown that imprecise encoding of spatial locations in a visual delayed response task is one factor that increases specifically the amount of confident errors in patients with schizophrenia (Mayer and Park, 2012).

The N2pc is also sensitive to the number of items presented in visual object tracking and enumeration tasks (Drew and Vogel, 2008; Mazza and Caramazza, 2011; Ester et al., 2012). These findings have been interpreted in terms of an attentional mechanism that allows the visual system to individuate targets from one another in order to make them available for further cognitive operations – a process that might be relevant for successful WM encoding as well. However, in the context of the visual change detection task, findings concerning the effect of set-size on the N2pc amplitude have been mixed (McCollough et al., 2007; Ikkai et al., 2010; Störmer et al., 2013; Adam et al., 2018). To further specify the role of attention-based object individuation for efficient WM encoding, further studies could manipulate the number of items to-be-encoded and take into account different response types as demonstrated in the present study.

Lastly, the exploratory analyses did not reveal effects of response type on N1pc or P2pc components. Consistent with a previous study (Störmer et al., 2013) this finding suggests that deficits in early visual stimulus processing did not contribute to WM encoding failures in our sample of young adults. However, this finding does not exclude that mechanisms that determine the efficiency of early visual processing and that are reflected for example by processing speed (Wiegand et al., 2014) rather than accuracy might explain failures of WM encoding.

### The Contribution of Failures in WM Encoding and Maintenance to WM Capacity Limitations

Consistent with our previous findings (Mayer and Park, 2012) the percentage of IC responses but not the percentage of IN responses correlated negatively with WM capacity estimates. These correlational findings further support the functional dissociation of confident and not-confident errors in the delayed orientation-discrimination task as indicated by the ERP results. Moreover, these findings suggest that the degree to which participants were able to use attentional mechanisms to efficiently encode the object orientations into WM was related to their individual WM capacity. These findings are consistent with the previous evidence that individual differences in attention-control capabilities contribute to variation in visual WM capacity (Vogel and Machizawa, 2004; Vogel et al., 2005; Fukuda and Vogel, 2009, 2011; Robison et al., 2018) – a finding that has in cognitive models been explained by shared limited resources of WM and attention (for a critical overview see Oberauer, 2019). However, the resource assumption does not necessarily predict that the capacity limit of WM correlates with every component of attentional capacity to the same degree. Indeed, within the mathematical framework provided by the theory of visual attention (Bundesen, 1990) it has been shown that perceptual processing speed, thought to depend on the attentional biases of the observer during WM encoding, and the capacity limit of visual WM reflect distinct processing resources – each associated with separable ERP markers during WM encoding (i.e., N1) vs. WM maintenance (i.e., CDA; cf. Wiegand et al., 2014). Note, however, that in the present study, individual WM capacity estimates did not correlate with individual differences in ERP amplitudes (i.e., the N2pc and the early CDA). On the one hand, we cannot exclude that this absence of the expected brain-behavior relationship was due to methodological constraints related, e.g., to the exclusion of participants from the EEG analyses, the unbalanced number of trials between different response types, and/or individual differences in perceptual processing speed (Wiegand et al., 2014; see section “Limitations”). On the other hand, this result is partly consistent with recent ERP studies showing that the N2pc elicited by processing of targets in the context of either visual search (Gaspar et al., 2016) or WM encoding (Feldmann-Wüstefeld and Vogel, 2019) was unrelated to individual differences in WM capacity (but see also Störmer et al., 2013). These two studies reported that individual differences in the ability to suppress unattended distractors was predictive of individual differences in WM capacity – a process that is indexed by the timing and amplitude of the distractor positivity (i.e., an enhanced positive-going ERP observed contralateral to task-irrelevant distractors in the same time window as the N2pc) rather than the N2pc. The exact attentional mechanisms that underlie IC responses in the present task and that may also contribute to the capacity limitation of WM as indicated by the behavioral findings, thus, warrant further clarification. The ability to suppress irrelevant information rather than attentional prioritization of relevant information might be a candidate process – a hypothesis that needs to be tested in future studies.

### Limitations

In the present study the to-be-encoded orientations were always presented for a constant time and, therefore, interindividual differences in encoding speed potentially influencing task performance and early visual ERPs (Wiegand et al., 2014) could not be taken into account. To minimize the influence of this critical confound, we implemented a stimulus presentation time that was longer than those previously established in the context of visual change detection tasks (Luck and Vogel, 1997). In addition, as indicated by the encoding control task, task performance and the distribution of response types were not significantly influenced by variations in encoding lengths (500 ms vs. 1,000 ms). Furthermore, two participants with very low performance in the sensory control task possibly due to deficits in the perceptual discrimination of the orientations were excluded from the study. Therefore, it seems unlikely that task performance and ERP amplitudes observed in the delayed orientation-discrimination task were driven to a large degree by differences in encoding speed. However, we cannot exclude that the short stimulus presentation time (150 ms) that was used in the change detection task in the present study led to an underestimation of WM capacity estimates in participants with low processing speed, thereby potentially confounding the correlational analyses.

An important limitation of this study was that the number of trials could not be balanced between different response types (CC = 353, CN = 98, IC = 87, and IN = 72 trials on average), which is inherent to the present paradigm. This resulted in a less noisy ERP waveform for CC trials and noisier waveforms for the remaining response types. To take differences in trial numbers into account, we analyzed mean amplitude, i.e., a relatively unbiased measure that is equally likely to produce larger or smaller values than the true value, while measures of peak activity more consistently produce larger values in noisier waveforms (Luck, 2014; Luck and Gaspelin, 2017). In addition, analyzing the data with LMMs (Kliegl et al., 2010) that explicitly model individual differences as random effects and thus take different amounts of data points per participant and condition into account (see Supplementary Material 5), we found ERP effects that were partially consistent with those derived from *t*-statistics supporting Hypothesis 1 but not Hypothesis 2.

Furthermore, it might be argued that the high number of correct responses in high-performing participants biased their confidence ratings also in incorrect trials (which were less frequent in these participants, due to their high rate of correct trials). This, in turn, might have led to increased CDA amplitudes for these participants, which might have confounded CDA amplitude differences between trial types observed across all participants. To assess this possibility, we compared the distribution of response types between high-performing and low-performing participants (Supplementary Material 2). The analyses revealed that high- and low-performing participants gave significantly more CC than CN responses, but for incorrect responses, the distribution of confident and not-confident responses was similar and this pattern was observed for both groups. These different distributions of confident and not-confident responses for correct and incorrect responses are not consistent with an overall response bias toward confident responses in high-performing participants. Thus, the present findings do not suggest that condition-specific CDA amplitude differences were confounded by indirect, performance-dependent effects on subjective confidence.

Lastly, three participants with overall high WM accuracy were excluded due to a lack of sufficient numbers of error trials. This may have reduced between-person variability in WM performance in the EEG sample. In addition, five further participants were excluded due to low trial numbers after artifact rejection. The resulting decrease of our sample size may have affected ANOVA and correlational results. For these reasons, we think that our exploratory results need to be interpreted with caution and that replications with larger samples and paradigms that focus on the analysis of very early perceptual processes during WM encoding are needed.

## Conclusion

Taken together, by analyzing temporally highly resolved electrophysiological measures depending on different response types in a delayed orientation-discrimination task, we demonstrated that failures in WM encoding and WM maintenance both contribute to limitations in WM performance in healthy adults. These electrophysiological findings underscore the relevance of distinguishing different types of responses in WM tasks in order to understand WM failures at different phases of processing. These findings have also important implications for understanding the sub-processes underlying WM decline, for example in older persons or in psychiatric patients. For instance, by analyzing different response types in a delayed response task, we have previously demonstrated that failures of WM encoding contribute to the severe WM deficit observed in schizophrenia (Mayer and Park, 2012; Mayer et al., 2014, 2018) – a core cognitive impairment that has been traditionally attributed to failures of WM maintenance (Goldman-Rakic, 1994). Given the convergent behavioral, electrophysiological, and neurocomputational evidence, we argue that analyzing temporally highly resolved electrophysiological measures depending on different response types can prove useful for investigating failures of WM sub-processes in healthy participants as well as WM impairments across different populations.

## Data Availability Statement

The datasets generated for this study are available on request to the corresponding author.

## Ethics Statement

The studies involving human participants were reviewed and approved by the Ethical Board of the Medical Faculty, Goethe University, Frankfurt. The participants provided their written informed consent to participate in this study.

## Author Contributions

JM and CF developed the conception and study design, performed the data analysis and interpretation, and wrote the manuscript. BP made substantial contributions to the study design and programming of the experiments. JM performed the data collection. SK made substantial contributions to EEG data acquisition and analysis. SK and BP provided critical revisions. All the authors approved the final version of the manuscript for submission.

## Conflict of Interest

The authors declare that the research was conducted in the absence of any commercial or financial relationships that could be construed as a potential conflict of interest.

## Footnotes

1 For comparison with our previous clinical studies (Mayer and Park, 2012; Mayer et al., 2014, 2018), IQ was measured with the Mehrfachwahl-Wortschatz-Intelligenztest (Lehrl, 1999; the German version of the National Adult Reading Test), which is an established measure for estimating premorbid IQ in clinical samples.

2 The orientation-discrimination paradigm was used because by manipulating the demands on WM precision rather than WM load (i.e., by varying the degree to which the probed bar in the test array is rotated relative to the corresponding bar in the memory array), this task has been shown to produce higher errors rates (Machizawa et al., 2012) than the typical change detection task where participants are asked to indicate if a change has occurred or not (e.g., McCollough et al., 2007).

3 Hit rate, the proportion of trials with a change on which participants report that an item changed; correct rejection rate, the proportion of trials without a change on which participants correctly report that an item did not change.
