# Supplementary material for: An Electrophysiological Dissociation of Encoding vs. Maintenance Failures in Visual-Spatial Working Memory
Source: Front Psychol. 2020 Mar 24;11:522. doi: 10.3389/fpsyg.2020.00522 (PMC7105797; doi:10.3389/fpsyg.2020.00522)
Supplement: Supplementary file 1 [file Data_Sheet_1.docx]

Supplementary Material

# Supplementary Data

## Supplementary Material 1. ERP results at individual posterior electrode pairs

N2pc and CDA components are usually measured at parietal-occipital electrodes and averaging across electrode pairs has emerged as a standard procedure (Luria et al., 2016). In the present study, we averaged across six posterior electrode pairs (P3/P4, P5/P6, P7/P8, PO3/PO4, PO7/PO8, O1/O2). To assess whether contralateral effects occurred at all electrode pairs, which is a prerequisite for averaging across electrode pairs, we explored contralateral difference waves at individual electrodes pairs. As an example, Supplementary Figure 1 shows the grand-averaged waveforms time-locked to memory array onset for correct/ confident responses (for which the largest number of trials was available) separately at each electrode pair. Visual inspection indicates that contralateral effects occurred at each posterior electrode pair and that they were most pronounced at PO7/PO8.


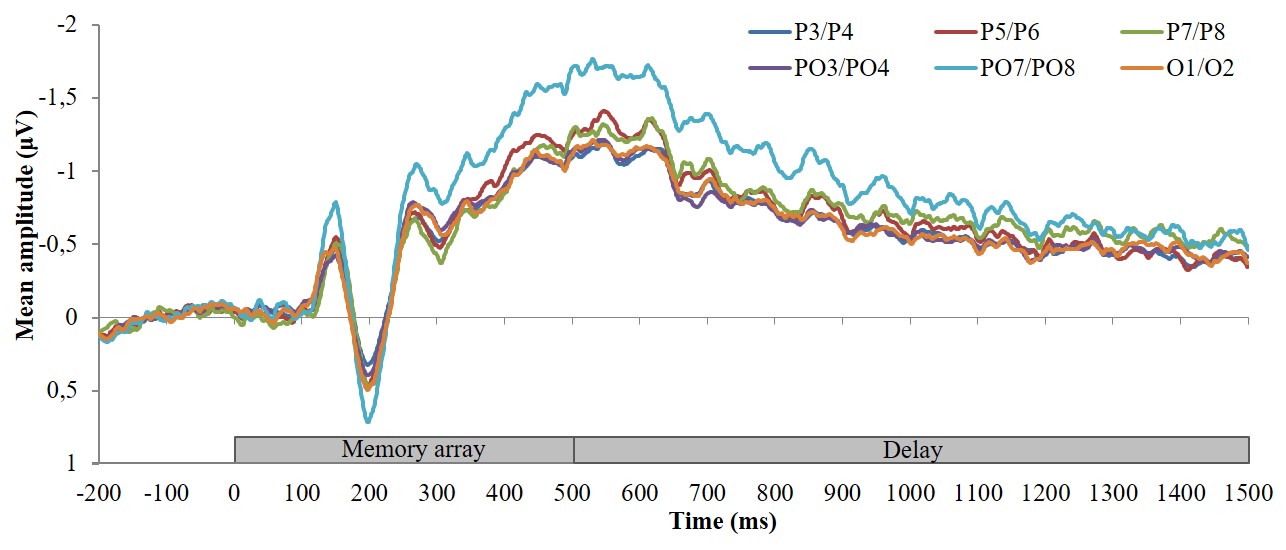


**Supplementary Figure 1.** Contralateral minus ipsilateral difference waveforms for correct/ confident trials at individual posterior electrode pairs (P3/P4, P5/P6, P7/P8, PO3/PO4, PO7/PO8, O1/O2)

To test whether lateralized ERP components differed between posterior electrode pairs, repeated-measures 2x2x6 ANOVAs were calculated separately for the N2pc (230-290 ms), early CDA (400-1000 ms), and late CDA (1000-1500 ms) components with the factors response correctness (correct vs. incorrect), confidence (confident vs. not-confident), and electrode pair (P3/P4, P5/P6, P7/P8, PO3/PO4, PO7/PO8, O1/O2).

For the N2pc, the ANOVA revealed no significant effect of electrode pair on the N2pc amplitude (see Supplementary Table 1). Furthermore, 2-way interactions between the factors electrode pair and response correctness and between the factors electrode pair and response confidence as well as the 3-way interaction were not significant.

For the early CDA component, there was a significant effect of electrode pair, however 2-way and 3-way interactions were not significant (see Supplementray Table 1). When leaving PO7/PO8 out, the effect of electrode pair on the amplitude of early CDA component was not significant [*F*(2.85, 99.77) = 1.66, *p* = .18]. These findings are consistent with the literature showing that the CDA is usually most pronounced at PO7/PO8 (Luria et al., 2016). However, the effects of response accuracy and response confidence did not differ between electrode pairs.

For the late CDA, there was neither a significant effect of electrode pair, nor significant 2-way (correctness x electrode pair, confidence x electrode pair) or 3-way interaction effects (see Supplementary Table 1).

Taken together, these findings demonstrate that contralateral effects occurred at all posterior electrode pairs, making it thus valid to average across these electrode pairs in the main analysis.

**Supplementary Table 1.** Results of the 2 x 2 x 6 ANOVAs^1^

| **ERP component** | **Main effect electrode pair** | **2-way interaction**  **electrode pair x correctness** | **2-way interaction**  **electrode pair x confidence** | **3-way interaction** |
| --- | --- | --- | --- | --- |
| N2pc | *F*(2.98, 104.27)  = 1.7, *p* = .17 | *F*(3.01, 105.49)  = .32, *p* = .81 | *F*(5, 175)  = 1.56, *p* = .17 | *F*(3.30, 115.32)  = .52, *p* = .69 |
| Early CDA | *F*(3.76, 131.45)  = 8.08, *p* < .001,  *ε^2^* = .19 | *F*(3.95, 138.27)  = .27, *p* = .90 | *F*(5, 175)  = 1.31, *p* = .26 | *F*(5, 175)  = .81, *p* = .54 |
| Late CDA | *F*(3.16, 110.74)  = .96, *p* = .42 | *F*(5, 175)  = .39, *p* = .86 | *F*(3.94, 137.87)  = .45, *p* = .77 | *F(*5, 175)  = 2.01, *p* = .08 |

^1^Greenhouse-Geisser correction was used for ANOVAs where sphericity was violated.

# Supplementary Material 2. Percentages of response types in the EEG delayed orientation-discrimination task for high- and low-performing participants

It might be argued that the high number of correct responses in high-performing participants biased their confidence ratings also for incorrect trials (which were less frequent in these participants, due to their high rate of correct trials). This, in turn, might have led to increased CDA amplitudes for these participants, which might have confounded CDA amplitude differences between trial types observed across all participants.

To assess this possibility in our sample, we divided the sample into high-performing (*N* = 22, *M* = 82.39, *SD* = 5.61) and low-performing (*N* = 22, *M* = 69.13, *SD* = 3.83) participants using a median split based on the mean accuracy in the delayed orientation-discrimination task, and compared differences in the distribution of confident and not-confident responses for correct and incorrect responses between groups. The distributions of response types for high-performing participants and low-performing participants are shown in Supplementary Table 2. Descriptively, for high-performing participants, about 50% of incorrect responses were given with high confidence. In contrast, for correct responses about 80% were given with high confidence and 20% were given with low confidence. These different distributions of confident and not-confident responses for correct and incorrect responses do not suggest an overall response bias towards confident responses in high-performing participants.

**Supplementary Table 2.** Results for high- and low-performing participants

| **Group** | **CC** | **CN** | **IC** | **IN** |
| --- | --- | --- | --- | --- |
| HP | 66.16% (13.75) | 16.38% (12.05) | 8.50% (5.25) | 8.96% (3.98) |
| LP | 52.93% (9.27) | 16.14% (8.95) | 17.66% (6.15) | 13.27% (5.05) |

Mean percentages (SD) are shown. CC = correct/ confident, CN = correct/ not-confident, IC = incorrect/ confident, IN = incorrect/ not-confident.

To statistically assess group differences in the distributions of response types, we calculated a repeated- measures 2x2x2 ANOVA with the factors correctness (correct vs. incorrect), confidence (confident vs. not-confident) and group (high- vs. low-performing participants). The ANOVA revealed a significanct 3-way interaction effect [*F*(1,42) = 11.99, *p* < .01, ɛ^2^= .22] indicating that the interaction between the factors correctness and confidence differed between groups. This was followed up by separate ANOVAs which revealed significant two-way interactions between the factors correctness and confidence, both for high-performing [*F*(1,21) = 121.78, *p* < .001, ɛ^2^= .85] and low-performing participants [*F*(1,21) = 182.29, *p* < .001, ɛ^2^= .90].

Follow-up paired *t*-tests showed that for high-performing participants, there was a significant difference in the percentage of confident and not-confident responses for correct trials [*t*(21) = 9.25, *p* < .001, Cohen’s *d* = 1.97] but no significant difference in the amount of incorrect/ confident and incorrect/ not-confident responses [*t*(21) = -.29, *p* = .78, Cohen’s *d* = 0.06, Bonferroni corrected statistical threshold for two tests: *p* = .025]. Similarily, for low-performing participants, there was a significant difference in the percentage of confident and not-confident responses for correct trials [*t*(21) = 9.68, *p* < .001, Cohen’s *d* = 2.06] but no significant difference in the amount of incorrect/ confident and incorrect/ not-confident responses [*t*(21) = 1.94, *p* = .066, Cohen’s *d* = 0.41, Bonferroni corrected statistical threshold for two tests: *p* = .025].

Taken together, high-performing and low-performing participants gave significantly more correct/ confident than correct/ not-confident incorrect responses, but for incorrect responses, the distribution of confident and not-confident responses was similar, both for high- and low-performing participants. These different distributions of confident and not-confident responses across correct and incorrect responses are not consistent with an overall response bias towards confident responses in high-performing participants. Thus, the present findings do not suggest that condition-specific CDA amplitude differences were confounded by indirect, performance-dependent effects on subjective confidence.

# Supplementary Material 3. The effect of encoding length on the percentage of response types in the encoding control task

Supplementary Figure 2 depicts the percentage of type of responses in the encoding control task with encoding lengths of either 500 ms (A) or 1,000 ms (B). Visual inspection indicates that the distributions of response types (CC, CN, IC, IN) were similar for both encoding lengths. To test whether the percentages of different types of responses differed as a function of encoding length, we conducted a 2x4 repeated-measures ANOVA with the factors encoding length (500 ms vs. 1,000 ms) and type of response (CC, CN, IC, IN). Greenhouse-Geisser correction was used for ANOVAs where sphericity was violated. The ANOVA revealed a significant interaction between the factors response type and encoding length [*F*(3, 126) = 3.37, *p* = .021, *ε^2^* = .07], indicating differential effects of encoding length on the percentage of different types of responses. Post-hoc paired *t*-tests (two-tailed) suggested that the interaction effect was driven by small differences between encoding lengths in the percentages of CC [500 ms: 55.2%, 1,000 ms: 58.9%, *t*(42) = -2.56, *p* = .014, Cohen’s *d* = .39] and IC responses [500 ms: 13.8%, 1,000 ms: 11.8%, *t*(42) = 1.97, *p* = .06] rather than differences in the percentages of CN [500 ms: 19.4%, 1,000 ms: 18.3%, *t*(42) = 0.88, *p* = .38] or IN responses [500 ms: 11.6%, 1,000 ms: 11.1%, *t*(42) = 0.53, *p* = .60]. Given that shortening encoding length had no substantial effect on the distribution of responses types, we decided to implement an encoding length of 500 ms in the EEG orientation change detection task in order to keep the trial duration as short as possible.

**
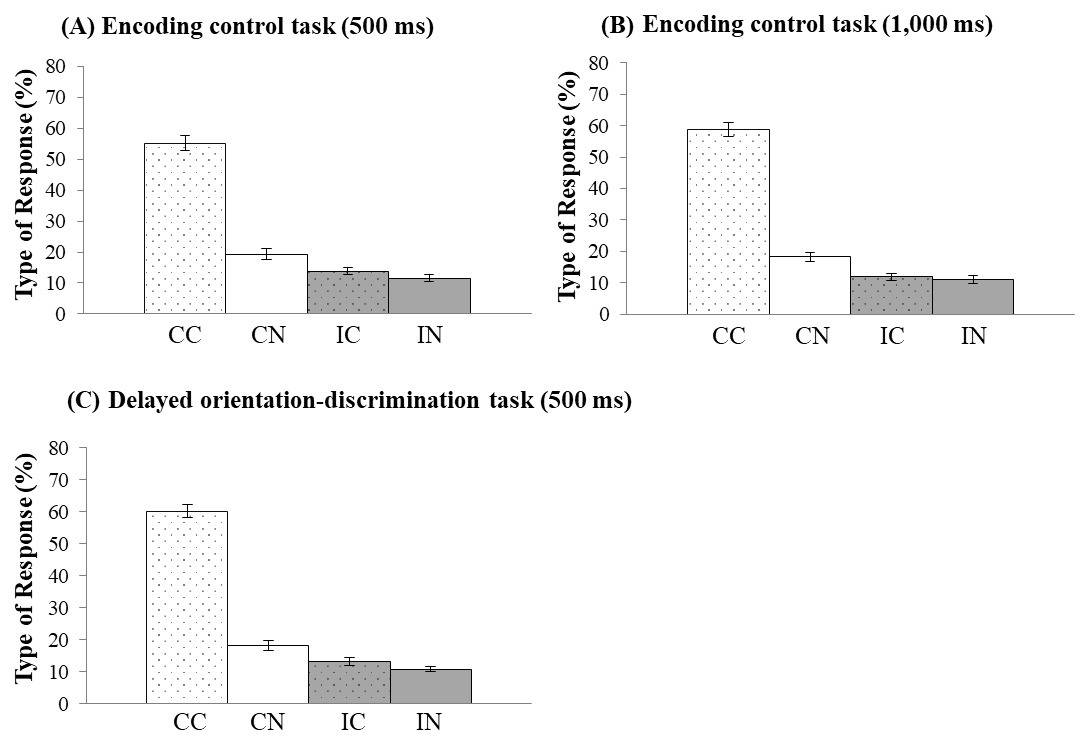
**

**Supplementary Figure 2.** Percentages of response types (A) in the encoding control task with encoding length of 500 ms, (B) with encoding length of 1,000 ms, and (C) in the EEG delayed orientation-discrimination task with encoding length of 500 ms. (N = 43, Data of one participant was missing due to technical problems during recording). Error bars represent the standard error of the mean. CC = correct/ confident, CN = correct/ not-confident, IC = incorrect/ confident, IN = incorrect/ not-confident.

# Supplementary Material 4. Percentages of response types in the EEG delayed orientation-discrimination task compared to the encoding control task

Encoding time was 500 ms in the EEG delayed orientation-discrimination task and either 500 or 1,000 ms in the encoding control task. To assess differences in the percentages of types of responses between tasks, we included only trials with encoding length of 500 ms in the analysis. The 2x4 ANOVA with the factors task (encoding control vs. delayed orientation-discrimination) and type of response (CC, CN, IC, IN) revealed a significant interaction between the two factors [*F*(2.09, 87.72) = 5.37, *p* = .006, *ε^2^* = 0.11, Greenhouse-Geisser corrected]. Post-hoc *t-*tests indicated that the percentage of CC responses was significantly higher in the EEG delayed orientation-discrimination task (60.2%) compared to the encoding control task [(55.2%), *t*(42) = 2.74, *p* = .009, Cohen’s *d* = .42] whereas the percentage of CN responses was significantly lower in the EEG task (15.7%) compared to the encoding control task [(19.4%), *t*(42) = -2.64, *p* = .011, Cohen’s *d* = .40 ]. In contrast, the percentage of incorrect responses did not differ between tasks [IC: *t*(42) = -.59, *p* = .558; IN: *t*(42) = -.72, *p* = .47] (Supplementary Figures 2A and 2C). These findings suggest that practice with the task due to performing the encoding control task in a session preceding the EEG measurement and/ or the high trial number in the EEG experiment increased the confidence with which correct responses were given but did not change the amount of incorrect responses.

# Supplementary Material 5. Reanalyses with linear-mixed effect models

Inherent to our paradigm, participant-specific variations in the amount of correct and incorrect responses and confidence ratings generated an unbalanced distribution of available data points (i.e., trials) across participants in each of the four response conditions. The lower number of trials in all other conditions compared to the correct/ confident condition [mean number of trials averaged across the 12 posterior electrodes: CC = 353 (*SD* = 75.03, range = 193-516), CN = 98 (*SD* = 52.91, range = 26-239), IC = 87 (*SD* = 41.30, range = 26-169), IN = 72 (*SD* = 30.84, range = 30-145)], thus, likely distorted effect estimates due to differences in signal-to-noise ratio between conditions. A suitable method to address this problem is the use of linear mixed-effect models (LMM; Kliegl et al. 2010; Bates et al., 2015). Namely, LMMs can account for variance traditionally treated as unexplained residuals in *t*-tests or ANOVAs by explicitly modeling individual differences as random effects.

Therefore, data was reanalyzed with LMMs. Mean amplitudes of single EEG segments - after pre-processing - were extracted for each of the three component’s time windows (i.e., N2pc, early CDA, late CDA) and served as the dependent variables in LMMs calculated with R (R Core Team, 2014) and the lme4 package (Bates et al., 2015). Planned contrasts between specified conditions (representing fixed effect terms) driven by our hypotheses were calculated (see Supplementary Table 3). Only random intercepts were allowed for participants, as the inclusion of random slope terms led to singularity warnings, indicating a model over-fit. Traditionally reported *p*-values as indicators for statistical significance are intentionally not provided by lme4 package, since there is no consensus on how to calculate meaningful denominator degrees of freedom in mixed designs (Bates et al., 2015). However, model outcomes include *t*-values that - due to our sample size of *N* = 36 and the large number of observations (i.e., single EEG segments) for each participant - effectively match *z*-statistics. Consequently, effect estimates two times larger than their standard error (i.e., absolute *t*-values of 2) can be interpreted as significant at the 5% level (Kliegl et al., 2010).

The findings derived from LMMs - which explicitly model individual differences as random effects and thus take different amounts of data points per participant and condition into account - were partially consistent with the results derived from planned *t*-tests reported in the main manuscript.

As shown in Supplementary Table 3, the N2pc amplitude was significantly reduced for incorrect/ confident responses compared to correct/ confident responses whereas early and late CDA amplitudes did not differ between these response types. These findings were consistent with the results derived from planned *t*-tests reported in the main manuscript supporting hypothesis 1. However, LMMs did not reveal a significant reduction of early and late CDA amplitudes for incorrect/ not-confident responses compared to incorrect/ confident responses. These findings were partially inconsistent with the findings based on *t*-statistics (which indicated a significant effect of response confidence on the early CDA for incorrect trials) and inconsistent with hypothesis 2.

Given these inconsistencies, we also explored CDA amplitudes for incorrect/ not-confident responses in comparison to correct/ confident responses assuming successful WM maintenance in the latter condition because CDA amplitude reductions have been shown for incorrect/ poor performance trials relative to correct/ good performance trials in the context of change detection tasks (McCollough et al., 2007; Adam et al., 2018). Note, this contrast was calculated in addition to the planned contrasts reported in the main manuscript. Consistent with previous findings, LMMs revealed that the amplitude of the late CDA was significantly reduced for incorrect/ not-confident responses compared to correct/ confident responses, which most likely reflects a failure of WM maintenance (McCollough et al., 2007; Adam et al., 2018). However, LMMs did not reveal a similar amplitude reduction for the early CDA. Given these inconsistencies, CDA reductions associated with incorrect/ not-confident trials reported here and in the main manuscript should be considered preliminary and replications with different and larger samples are needed.

**Supplementary Table 3.** Results of linear mixed-effect models

| **Component** | **Contrast** | **Estimate (*SE*)** | ***t*** | **Sign.** |
| --- | --- | --- | --- | --- |
| **N2pc** | Intercept | -0.59 (0.10) | -5.68 | * |
|  | CC vs. IC | 0.23 (0.11) | 2.07 | * |
| **CDA early** | Intercept | -1.02 (0.11) | -9.09 | * |
|  | CC vs. IC | 0.08 (0.10) | 0.83 | *ns* |
|  | Intercept | -0.98 (0.15) | -6.56 | * |
|  | IC vs. IN | 0.18 (0.13) | 1.38 | *ns* |
|  | Intercept | -1.03 (0.11) | -9.23 | * |
|  | CC vs. IN | 0.21 (0.11) | 1.96 | *ns* |
| **CDA late** | Intercept | -0.53 (0.11) | -4.10 | * |
|  | CC vs. IC | 0.08 (0.11) | 0.77 | *ns* |
|  | Intercept | -0.47 (0.15) | -3.21 | * |
|  | IC vs. IN | 0.21 (0.15) | 1.46 | *ns* |
|  | Intercept | -0.52 (0.12) | -4.48 | * |
|  | CC vs. IN | 0.24 (0.12) | 2.10 | * |

# References

Adam, K. C. S., Robison, M. K., and Vogel, E. K. (2018). Contralateral delay activity tracks fluctuations in working memory performance. *J. Cogn. Neurosci* 30, 1229-1240. doi: 10.1162/jocn_a_01233

Bates, D., Mächler, M., Bolker, B., & Walker, S. (2015). Fitting linear mixed-effects models using lme4. *J. Stat. Softw.* 67(1), 1–48. doi:10.18637/jss.v067.i01

Kliegl, R., Wei, P., Dambacher, M., Yan, M., and Zhou, X. (2010). Experimental effects and individual differences in linear mixed models: Estimating the relationship between spatial, object, and attraction effects in visual attention. *Front. Psychol.* 1, 1–12. doi:10.3389/fpsyg.2010.00238

Luria, R., Balaban, H., Awh, E., & Vogel, E.K. (2016). The contralateral delay activity as a neural measure of visual working memory. *Neurosci. Biobehav. Rev.* 62, 100–108. doi: 10.1016/j.neubiorev.2016.01.003

McCollough, A. W., Machizawa, M. G., and Vogel, E. K. (2007). Electrophysiological measures of maintaining representations in visual working memory. *Cortex* 43, 77–94.

R Core Team. (2014). R: A language and environment for statistical computing. Vienna, Austria: R Foundation for Statistical Computing. Retrieved from http://www.r-project.org/
